# Correction of severe valgus deformity of knee osteoarthritis with non-constrained total knee arthroplasty implant: A case report

**DOI:** 10.1016/j.ijscr.2018.10.080

**Published:** 2018-11-02

**Authors:** Dwikora Novembri Utomo, Ferdiansyah Mahyudin, Andre Yanuar, Lukas Widhiyanto, Kukuh Dwiputra Hernugrahanto

**Affiliations:** a Orthopedic and Traumatology Department, Faculty of Medicine, Universitas Airlangga/Dr. Soetomo General Hospital, Surabaya, Indonesia; b Santo Borromeus Hospital, Bandung, Indonesia

**Keywords:** Severe valgus deformity, No consensus, MCL shift, Non-constrained implant, Case report

## Abstract

**Introduction:**

Knee osteoarthritis with valgus deformity presents a surgical challenge that must be solved during total knee arthroplasty (TKA). In some case whereby ligament imbalance is found, constrained implant should be used. Unfortunately, the implant is not always readily accessible in some developing countries. The objective of this paper is to provide alternative solution to such case.

**Presentation of case:**

We reported a 71-year-old female patient with a painful and fixed valgus deformity of her right knee. Preoperatively, the patient’s right knee range of motion (ROM) was 10–145° of flexion with a 32° fixed valgus deformity. A constrained implant was not accessible. To balance the ligament, Medial Collateral Ligament (MCL) origin was shifted to superior and anterior. A non-constrained implant was used. The valgus deformity was corrected intraoperatively and ROM achieved 0–140° of flexion.

**Discussion:**

It is crucial that attention is given not only to the restoration of proper bony alignment but even more importantly to soft tissue balancing. By using the non-constrained implant, the cost can also be reduced and suitable for developing countries with limited coverage of state insurance.

**Conclusion:**

Three months after the surgery, the patient achieved stable and painless knee with 10–90° of flexion and complete correction of her valgus deformity.

## Highlights

• Knee osteoarthritis with severe valgus deformity is a challenging case.
• The correction usually requires the use of high-cost constrained knee implant.
• Non-constrained implant with ligament adjustment provide good outcome.

## 1 Introduction

Knee osteoarthritis with valgus deformity presents a surgical challenge that must be solved during total knee arthroplasty (TKA). This deformity (defined as a valgus angle equal to or greater than 10°) is observed in nearly 10% of patients undergoing TKA [1]. The valgus deformity in osteoarthritis knee is a continuing process developed by bone tissue remodeling and soft tissue contraction/elongation. Bone tissue alterations consist of lateral cartilage erosion, lateral condylar hypoplasia, and metaphyseal femur and tibial plateau remodeling. Soft tissue alterations are characterized by tightening of lateral structures such as lateral collateral ligament (LCL), posterolateral capsule (PLC), popliteus tendon (POP), hamstring tendons, the lateral head of the gastrocnemius (LHG) and iliotibial band (ITB). Some authors also described a posterior cruciate ligament (PCL) alteration in valgus knees, but in the literature, its influence in maintaining the deformity is not universally recognized. The described deformities can lead to a tibial external rotation and to a patellar lateral subluxation tendency [2]. Three grades of valgus deformity have been described. In grade I, the deviation is less than 10°, passively correctable with contracture of the lateral soft tissue but without elongation of the medial collateral ligament (MCL, 80% of cases). In grade II, the axial deviation ranges between 10° and 20°, the lateral structures are contracted and the MCL is elongated but functional (15% of cases). Grade III deformity is present in the remaining 5% of the patients; the axial deformity is greater than 20°, the lateral structures are tight and the medial stabilizers are not functional [1,3].

Valgus deformity in osteoarthritis, for this reason, is a challenge for the surgeon both for gap balancing and implant constrained choice. Several different surgical techniques have been described to perform TKA in valgus deformity; the aim of this article is to give an optional soft tissue balancing by re-tensioning MCL and using the non-constrained implant. This case report is written according to the recently published SCARE criteria as it used for supporting transparency and accuracy in publication of case-reports [4].

## 2 Presentation of case

Mrs. P is a 71-year-old, slightly obese Indonesian female with an atraumatic progressive painful deformity of her right knee. The patient had become limited to minimal house activity. She required a walker and/or wheelchair for independent mobility at home. She had difficulty to stand and could no longer climb stairs without assistance. Her pain was in proportion to her activities and was unresponsive to nonsteroidal anti-inflammatory drugs (NSAIDs) medication. She had no history of comorbidities related to her knee complaints.

On physical examination, the patient was found to be an alert, oriented female sitting in a wheelchair in no apparent distress. However, she had significant pain on her right knee when she tried to bear-weight. The skin over the right lower extremity was intact and there was no effusion, soft tissue swelling or erythema about the right knee. The right knee was held in a flexed posture (Fig. 1). The right knee range of motion (ROM) was relatively painless but limited to 10-145° of flexion with a 32° (Grade III) fixed valgus deformity.

**Fig. 1:**
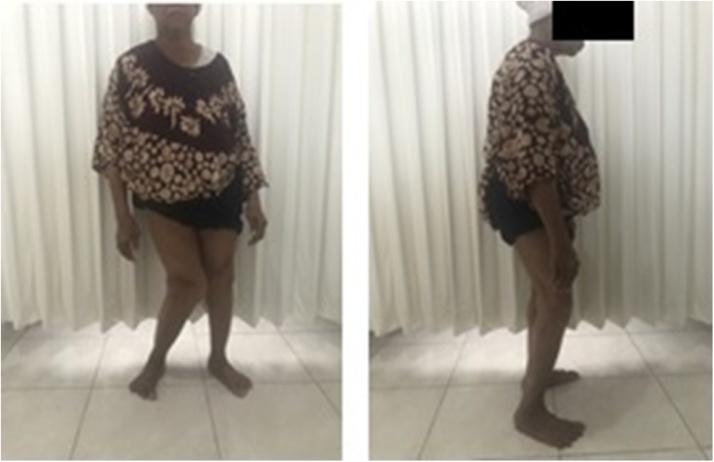
Patient’s clinical appearance (source: internal documentation).

Left knee also had 10° valgus deformity, however, the ROM of the left knee and both hips were painless within normal limits. X-ray demonstrated a significant valgus deformity of the right knee, erosion of lateral femoral condylar bone and severe tri-compartmental osteoarthritis (Fig. 2). After all of failed attempts of conservative options of treatment, the patient agreed for right total knee arthroplasty. The surgery was performed by the author.

**Fig. 2:**
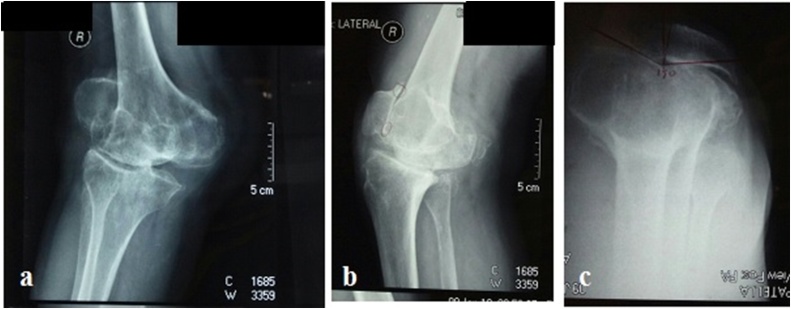
(a–c) Radiological imaging shows severe osteoarthritic valgus deformity knee (source: internal documentation).

Preoperatively, we had assessed the right knee with severe valgus deformity. We found that the lateral aspect was tight. Therefore, initially we decided to use constrained implant in this patient. Unfortunately, due to limited coverage of the state insurance in, the constrained implant was inaccessible. Therefore, we carried on with the surgery using standard non-constrained implant with addition of soft tissue balancing procedure.

The patient underwent a TKA with epidural anesthesia and intra-operative tourniquet. We performed a medial parapatellar approach. First, we performed the tibial cut, perpendicular to the anatomical axis and removed the smallest possible bone amount, especially from the lateral side. After finishing the bone cuts, we once again tried to assess the balance of the tissue. It was found that there was soft tissue imbalance due to the severe valgus deformity. The lateral ligaments were tight with loose medial ligaments as predicted preoperatively. Ideally, this grade III valgus deformity should be corrected with constrained implant. Therefore, as planned preoperatively, we did an alternative soft tissue procedure with non-constrained implant.

The MCL origin was cut together with the bone, such as in avulsion fracture. The origin was shifted superiorly in extension. Then, the balanced position was marked. The balancing was continued in flexion position which needs anterior shift of the already marked new position of MCL origin. The new MCL origin was fixed with 3 cortical screws, expecting a bone to bone healing (Fig. 3). ROM achieved intra-operatively was 0–140° of flexion with no valgus deformity. The patella was resurfaced with a 6 mm thickness polyethylene component. This patient used Medacta Evolis^®^ Primary Posterior Stabilized Implant TKA with an 11 mm polyethylene insert. Postoperative X-ray was shown in Fig. 4. The surgery took two hours since skin incision until wound closure. Blood lost was estimated about 500 ml. Postoperatively the patient's treatment included: routine DVT prophylaxis, gentle progressive active and active-assisted ROM and non-weight-bearing activities for 5 weeks until clinical union.

**Fig. 3:**
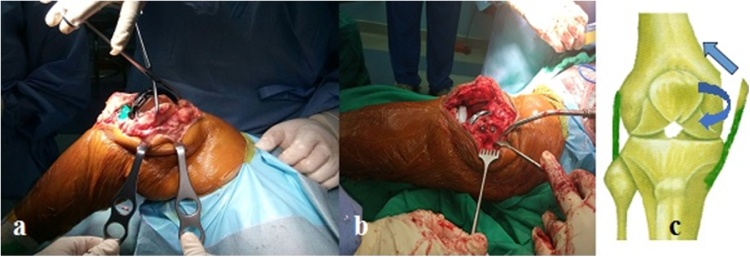
(a, b) Medial Collateral Ligament (MCL) origin was shifted to superior and anterior then fixed using 3 screws, (c) Illustration of the MCL origin shift (source: internal documentation).

**Fig. 4:**
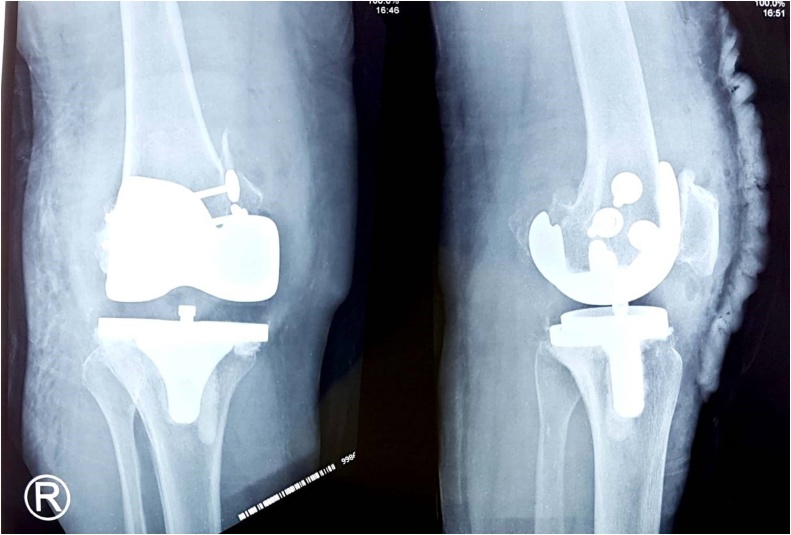
Post surgery result (source: internal documentation).

The patient had a good postoperative recovery and was discharged to a rehabilitation facility to continue her therapy. She returned to her home after a week post-surgery and walked independently in non-weight-bearing with a walker. Rehabilitation was continued, initially within the home and then outside the home. By three months post-TKA, the patient had achieved 10–90° of right knee flexion, with no recurrence of her valgus deformity (Fig. 5).

**Fig. 5:**
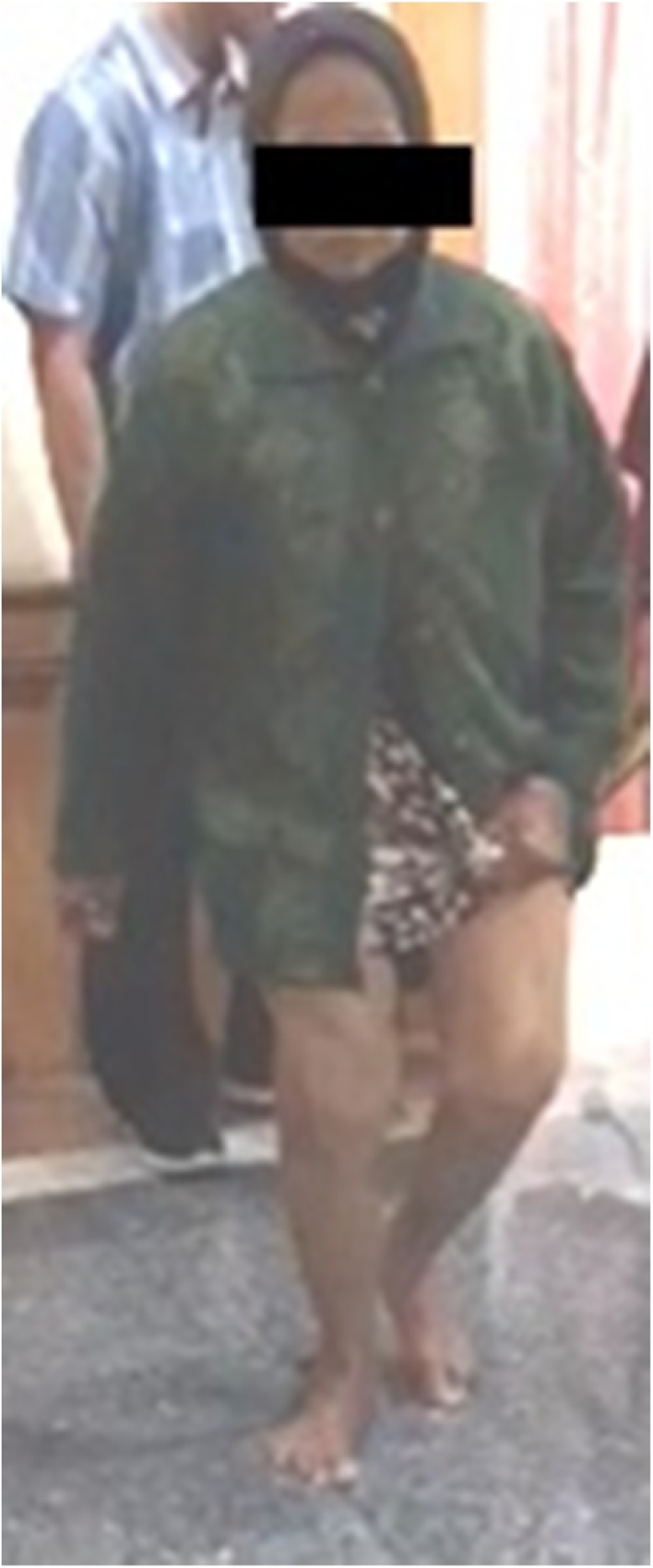
Clinical appearance 3 months following the surgery; stable while walking (source: internal documentation).

## 3 Discussion

Soft tissues are extremely important for the physiologic functioning of the knee joint [5]. Severe osteoarthritis is often associated with deformity and compromise of normal soft tissue. TKA is a highly successful procedure for the relief of painful arthritis [6]. However, when attempting to restore function and correct abnormalities in ROM and alignment by TKA, it is crucial that attention is given not only to the restoration of proper bony alignment but even more importantly to soft tissue balancing. Sufficient and good soft tissue balance will make the knee stable with non-contrained implant. However, insufficient or incorrect soft tissue balancing may result in the limitation in ROM, patellar mal-alignment, knee instability, premature mechanical failure of the TKA components and pain which result in the need of using contrained implant to address the instability issue [7]. There is no consensus regarding the sequence in which the structures about the knee should be released in the valgus knee [8,9]. Different authors tried to describe a lateral structure release sequence based on functional and anatomical consideration. If the release of the lateral structures does not sufficiently stabilize flexion and extension gaps, then the medial side of the joint should be addressed. Several techniques have been described for successfully and safely tightening the incompetent medial collateral ligament (MCL). Krackow et al. [3] described MCL advancement of the tibial side, and Krackow [10] described MCL mid-substance division and imbrication to equalize the joint gaps. Ranawat’s “pie-crusting technique” has been described for releasing the lateral structure with an inside-out procedure. In this technique, the tight lateral structures are palpated when the lamina spreaders are inserted and they are released performing multiple transverse incisions with a number 15 surgical blade [1].

Healy et al described recessing the origin of the MCL with a bone block from epicondyle [11]. Although these procedures are technically demanding and may affect ligament strength and isometricity, they may be necessary to equalize joint gaps to achieve a stable and durable result. Our early experience using this technique also showed stable and durable after 3 months following surgery (Fig. 5). Another important consideration in the management of valgus deformity is prosthesis selection with regard to the degree of component constrained. Ideally, this patient should have used constrained implant. However, in our country, the state insurance coverage was limited and therefore the implant became inaccessible. There are numerous type of implants ranging from non-constrained to constrained ones. Each type and design has its own degree of constrain, advantages, and disadvantages. Constrained is the limitation of motion between two bodies linked by a joint. There is a spectrum of constrained options available during TKA. Cruciate retaining (CR) prosthesis provides the least amount of constrained. Posterior stabilized (PS) knee prosthesis comes at the next level of constrained. It sacrifices the Posterior Cruciate Ligament (PCL) and provides posterior stability by virtue of a tibial polyethylene post, which engages in the intercondylar cam of femoral component. However, it provides little varus-valgus and rotational stability. Condylar constrained knee (CCK) is at the next level of constrained prostheses. It is a semi constrained, non- linked implant, which confers varus-valgus as well as some rotational stability. Hinge knee prosthesis is a linked constrained device, which provides the highest level of component-to-component constrained, and therefore, confers coronal plane, sagittal plane, as well as rotational stability. The primary goal of total knee arthroplasty is to provide a pain free stable joint, to restore knee kinematics, and alignment with good component fixation, which translate into successful functional and clinical outcome. As we know, if proper soft tissue balance is restored, a minimally constrained implant can be used. Therefore, after we made a good soft tissue balance, we decided to use PS knee prosthesis [12]. By using the non-constrained implant, the cost also can be very significantly reduced and suitable for our country’s limited coverage of state insurance.

## 4 Conclusion

The valgus knee presents a surgical challenging problem that must be addressed during total knee arthroplasty. Both bone and soft-tissue deformities complicate restoration of proper alignment, positioning of components, and attainment of joint stability. The variables that may need to be addressed include lateral femoral condyle or tibial plateau deficiencies secondary to developmental abnormalities, and/or wear and laxity of the medial collateral ligament. We found that shifting MCL origin proximally could give stable result after 3 months following surgery and non-constrained implant work well by using this technique. Further evaluation with more number of cases and longer term of evaluation is needed to assess the effectivity and survival using this technique.

## Conflicts of interest

The authors have no conflict of interest to declare.

## Funding source

There is no specific grant from funding agencies in the public, commercial, or not-for-profit sectors.

## Ethical approval

Approval to publish case report is waived by the institution.

## Consent

Written informed consent was obtained from the patient for publication of this case report and accompanying images. A copy of the written consent is available for review by the Editor-in-Chief of this journal on request.

## Author contribution

Dwikora Novembri Utomo, surgeon: performed the literature review and data collection, designed the manuscript.

Ferdiansyah Mahyudin, surgeon: performed the literature review and data collection, designed the manuscript.

Andre Yanuar, surgeon: contributed to the manuscript writing and data collection.

Lukas Widhiyanto, surgeon: data collection and designed the manuscript.

Kukuh Dwiputra Hernugrahanto: contributed to the manuscript writing and data collection.

## Registration of research studies

We have reported a single case with no requirement for registry.

This manuscript only describes observational study in patient.

## Guarantor

Dwikora Novembri Utomo.

Ferdiansyah Mahyudin.

Andre Yanuar.

Lukas Widhiyanto.

Kukuh Dwiputra Hernugrahanto.

## Provenance and peer review

Not commissioned, externally peer reviewed.
